# Are sucrose transporter expression profiles linked with patterns of biomass partitioning in *Sorghum* phenotypes?

**DOI:** 10.3389/fpls.2013.00223

**Published:** 2013-06-26

**Authors:** Ricky J. Milne, Caitlin S. Byrt, John W. Patrick, Christopher P. L. Grof

**Affiliations:** ^1^School of Environmental and Life Sciences, University of Newcastle, NewcastleNSW, Australia; ^2^Australian Research Council Centre of Excellence in Plant Cell Walls, Waite Campus, University of AdelaideAdelaide, SA, Australia

**Keywords:** expression profiling, *Sorghum*, source–sink pathway, sucrose transporters, sucrose storage

## Abstract

*Sorghum bicolor* is a genetically diverse C_4_ monocotyledonous species, encompassing varieties capable of producing high grain yields as well as sweet types which accumulate soluble sugars (predominantly sucrose) within their stems to high concentrations. Sucrose produced in leaves (sources) enters the phloem and is transported to regions of growth and storage (sinks). It is likely that sucrose transporter (SUT) proteins play pivotal roles in phloem loading and the delivery of sucrose to growth and storage sinks in all *Sorghum* ecotypes. Six *SUTs *are present in the published *Sorghum* genome, based on the BTx623 grain cultivar. Homologues of these *SUTs* were cloned and sequenced from the sweet cultivar Rio, and compared with the publically available genome information. SbSUT5 possessed nine amino acid sequence differences between the two varieties. Two of the remaining five SUTs exhibited single variations in their amino acid sequences (SbSUT1 and SbSUT2) whilst the rest shared identical sequences. Complementation of a mutant *Saccharomyces* yeast strain (SEY6210), unable to grow upon sucrose as the sole carbon source, demonstrated that the *Sorghum* SUTs were capable of transporting sucrose. *SbSUT1*, *SbSUT4*, and *SbSUT6* were highly expressed in mature leaf tissues and hence may contribute to phloem loading. In contrast, *SbSUT2* and *SbSUT5* were expressed most strongly in sinks consistent with a possible role of facilitating sucrose import into stem storage pools and developing inflorescences.

## INTRODUCTION

The storage of organic carbon as non-structural carbohydrates by plants is of biological and commercial interest. In this context, many varieties exist within the genetically diverse *Sorghum bicolor* species ranging from grain *Sorghum* types which store large amounts of starch within their grains to sweet *Sorghum* types which accumulate sucrose/hexoses within their stems. Sweet *Sorghum* cultivars are capable of accumulating soluble sugars up to 60% of their internode dry weight (Hoffmann-Thoma et al., 1996). The Rio cultivar can accumulate three times the amount of sugar (total glucose, fructose, and sucrose g/kg stem tissue) in mature stems compared to grain *Sorghum* cv. BTx623 (Murray et al., 2008). For these reasons, sweet *Sorghum*, a C_4_ monocotyledonous plant with high yield potential, is regarded as an ideal feedstock to provide sugar for bioethanol production. Higher sugar lines are preferred for the production of “first generation” bioethanol. A high sugar variety may yield 500 g of sugar per kg of stem dry weight, and total soluble sugar yields can reach 10 t ha^-^^1^ (Zhao et al., 2009). These yields equate to theoretical ethanol yields of up to 5414 L ha^-^^1^ (Zhao et al., 2009). However, higher sugar, and hence ethanol yields per hectare may be achievable through selective breeding and/or genetic transformation of *Sorghum*.

During sugar accumulation within stems, sucrose produced in photosynthetic source leaves is transported within phloem sieve element-companion cell (SE-CC) complexes to an array of sinks (non-photosynthetic organs) comprising developing vegetative and reproductive organs (growth sinks) as well as the stem storage sink. Within growth sinks carbohydrates are invested primarily into the biosynthesis of cellular structures. In contrast, elongating and mature internodes of cv. Rio accumulate sucrose within vacuoles, cytosols, and apoplasmic spaces of their storage parenchyma cells (Lingle, 1987).

In the C_4_ species maize (*Zea mays*), closely related to *Sorghum*, sucrose loading of SE-CC complexes occurs apoplasmically (Slewinski et al., 2009). It is assumed that a similar pathway of phloem loading of sucrose is followed in *Sorghum*. In stems of sugarcane and *Sorghum*, sucrose is transferred radially from their SE-CC complexes into storage parenchyma cells. Intracellular compartmentation of stored sucrose in *Sorghum* is presumed to be similar to that of sugarcane. Here, the bulk of sucrose accumulates within vacuoles of their storage parenchyma cells to concentrations that equal or exceed sucrose concentrations of the phloem sap. Thus the possibility of a concentrating step is invoked. Since the pathway of phloem unloading follows a symplasmic pathway in sugarcane stems (Jacobsen et al., 1992), any concentrating step must be localized to tonoplasts of their storage parenchyma vacuoles. Inconsistent with this conclusion is the finding that sucrose transport into isolated vacuoles of sugarcane stems occurs by facilitated diffusion (Williams et al., 1990; Preisser and Komor, 1991). However, whether an energy-dependent transport step operates in parallel with facilitated diffusion into vacuoles, as reported for sugar beet (Saftner et al., 1983), remains to be resolved for sugarcane. In the case of *Sorghum*, the phloem unloading pathway of sucrose into stem storage parenchyma cells appears to include an apoplasmic component (Tarpley and Vietor, 2007) and hence an additional reliance on movement across plasma membranes arranged in series with tonoplast transport.

Import of sucrose into cells across their plasma membranes is mediated by sucrose transporters (SUTs). SUTs are energy-dependent trans-membrane proteins which co-transport sucrose and protons in the same direction, in a 1:1 stoichiometric ratio (Lalonde et al., 2004). Therefore, *Sorghum* SUTs are of interest because they may play key roles in apoplasmic phloem loading of sucrose in source leaves and apoplasmic unloading of sucrose into stem storage sinks (see above). SUTs are known to function in phloem loading of maize source leaves (Slewinski et al., 2009) but the role of SUTs in stem storage is less certain. Here the final sucrose concentration within stems can be a balance between import and remobilization to provide a supplementary source of organic carbon to support grain filling when leaf photosynthesis has been depressed by stressful conditions (Blum et al., 1994, 1997). However, remobilization of stem reserves in a number of *Sorghum* cultivars has been reported to be minimal under favorable environmental conditions (Gutjahr et al., 2013).

Here we investigate the expression of *Sorghum* SUTs in source and sink organs during vegetative growth and at anthesis in two cultivars of *Sorghum*, cv. BTx623 and cv. Rio. These two cultivars exhibit very different phenotypes, with cv. BTx623 being of short stature and producing a large grain head. In contrast, cv. Rio produces a small panicle with fewer grains, but may grow to a height of 3 m with a stout culm for sugar storage. Differences in *SUT* expression between cultivars may correlate with phloem loading, long distance transport, and ultimately partitioning of sucrose to reproductive sinks in cv. BTx623 or stem sinks in cv. Rio. Complementation of the deficient *Saccharomyces cerevisiae *SEY6210 strain by *Sorghum* SUTs is also explored as a first step toward detailed functional characterization of these transporters.

## MATERIALS AND METHODS

### PLANT GROWTH CONDITIONS

Seeds of the *Sorghum* cultivars Rio and BTx623 were germinated and grown in 10 L pots containing a soil mixture consisting of two parts coarse sand, one part coco peat, and one part perlite, under glass house conditions with temperatures maintained at 25.5 ± 1.5°C during the day, and 15.5 ± 0.5°C during the night. Plants were exposed to a photoperiod of 14-h light and 10-h dark cycle with supplementary lighting provided by tungsten incandescent lamps. Seedlings were thinned to one per pot at 1-week post germination. Pot water levels were maintained at field capacity with a programmable drip irrigation system delivering water to each pot for two min, three times per day. Osmocote exact slow release fertilizer (Scotts Australia Pty Ltd, Sydney, NSW, Australia) was applied at a rate of 20 g per pot 2-weeks post germination and was supplemented with liquid fertilizer (Wuxal Liquid Foliar Nutrients; AgNova Technologies Pty Ltd, Eltham, VIC, Australia) at fortnightly intervals. Nitrogen (N), phosphorus (P), and potassium (K) ratios for Osmocote exact were 15N, 3.9P, and 9.1K.

### HARVESTING PLANT MATERIAL

All plant samples were snap frozen in liquid nitrogen immediately following harvest. During the vegetative growth phase, cv. BTx623 (grain) and cv. Rio (sweet) were destructively harvested approximately 60 and 90 days after germination, respectively. Material harvested for analysis was a sink leaf (expanding leaf fully enclosed within leaf sheaths); source leaf (youngest fully expanded leaf), internode 2 (elongated internode; numbered acropetally), and internode 5 (elongating). At anthesis, cv. BTx623 and cv. Rio were harvested approximately 103 and 140 days after germination, respectively. The flag leaf and leaf 7 (numbered acropetally), the flag internode, internode 2 and whole inflorescences were harvested. Additional samples were taken for detailed analysis of SUT expression. These were upper portion (5 cm) of the flag internodes and inflorescences separated into spikelets, anthers, and rachis branches.

### ISOLATION OF TOTAL RNA

Tissue samples were cryogenically ground in stainless steel grinding jars cooled on dry ice with a cooled stainless steel ball bearing agitated for 1 min at 30 Hz using a Retsch TissueLyser II (QIAGEN, Chadstone Centre, VIC, Australia). Total RNA was isolated from 100 mg of ground material. Leaves were extracted using the plant RNeasy^®^ kit (QIAGEN) whilst stems and inflorescences were extracted using the plant RNA reagent (Life Technologies, Mulgrave, VIC, Australia). Digestion of contaminating genomic DNA was performed post RNA isolation using the Ambion^®^ TURBO^TM^ DNase kit (Life Technologies). RNA isolation and genomic DNA digestions were performed according to the manufacturer’s instructions.

### SYNTHESIS OF cDNA

Complementary DNA (cDNA) was synthesized from 1 μg of RNA using the Thermoscript^®^ first strand cDNA synthesis kit (Life technologies) with an oligo d(T) primer, at an extension temperature of 60°C, according to the manufacturer’s instructions.

### CLONING FULL-LENGTH GENES

Full-length coding DNA fragments of each *Sorghum* *SUT* was cloned from cDNA by polymerase chain reaction (PCR) using Fermentas 2xMM (ThermoFisher, Scoresby, VIC, Australia) spiked with 1 μL Fermentas Pfu polymerase (ThermoFisher) using gene specific primers (**Table 1**). PCR cycling conditions were 95°C for 10 min followed by 35 cycles of 95°C for 30 s, 55°C for 30 s, 72°C for 2 min (2 min 20 s for *SbSUT2*). Amplified products were cloned into the pGEM-t easy vector (Promega, Sydney, NSW, Australia) and at least three clones were sequenced from separate cDNA samples. *SUTs* were then amplified from plasmids using the Stratagene Pfu Ultra II polymerase (Integrated Sciences, Chatswood, NSW, Australia) by primers incorporating restriction sites at the start and stop codons as shown in **Table 1** and recommended cycling profile using a 55°C annealing temperature. Products were digested with corresponding FastDigest^®^ Fermentas restriction enzymes (ThermoFisher), as were the yeast expression vectors. *SUTs* were then ligated into pDR195 (*SbSUT5* and *SbSUT6*) or pDR196 (Rentsch et al., 1995).

**Table 1:** Primer sets used for PCR amplification of *Sorghum* *SUT*s. Restriction site sequences are underlined.

Gene	Forward primer (5′–3′)	Reverse primer (5′–3′)
**Full-length primers**
*SbSUT1*	GTCGTCCCGTACGTGTGC	ATCTTGCACGGTTGGGTTT
*SbSUT2*	CCGCAGCGACACCTACAC	AATGGCAAAATGGGGCTAAGT
*SbSUT3*	CTCCACACCTCTCCGGTTT	CGACAGTAGTGGTTGATCG
*SbSUT4*	TCAAAGCAACTCAGCGATTC	AGCTGCAACTCTTCCAAAGC
*SbSUT5*	GTAGCCATGGACGGTGGTG	CCGCCTGGCGATAGATAGAT
*SbSUT6*	CGTTCCTGCTCCTCTCACTC	TGGATTTCCGATCATCCACT
**Restriction cloning primers**
*SbSUT1*	CTCGCGGAATTCATGGCTCGCGGCGA	GGCCGTGTCGACTCAGTGGCCGCCCG
	EcoRI	sall
*SbSUT2*	GGCGCGGTCGACATGGACGCCGGCACC	TTGGGCAGTCGACTCAGCCAAATCCATGG
	sall	XhoI
*SbSUT3*	CCGGTTGAATTCATGGCTGCTGATGGC	CTGGACCTCGAGTCAATGGCCTCCTC
	EcoRI	XhoI
*SbSUT4*	CCGTGAGAATTCATGCCGCCGCGCAC	GTAATGGTCGACATTATCGGTGCGTGC
	EcoRI	sall
*SbSUT5*	AATTCGAGCGGCCGCATGGACGGTGGTGAC	GCGATAGGATCCTCAGTGGCCGCCGC
	NotI	BamHI
*SbSUT6*	GCCCGGCGGCCGCATGGACGACGGTGAC	CCTGGAGGATCCTCAACAGTGGCCGC
	NotI	BamHI
**qPCR primers**
*SbSUT1*	GTGCTCCTGTAATCTTTGTGTCC	ACTATACTGCACATTGATTGATCG
*SbSUT2*	GCACATGCATTGAATGAACC	TTCGCATTTGGAAATTCCTC
*SbSUT3*	GGCCGGATCAAACAAGAT	GGCATTGCGAAGGAATGA
*SbSUT4*	CGATCCATGATGATGTCCAG	GTTCCAGGCCTTGCTGTC
*SbSUT5*	CCCGTAGTGTTGCGGAGTC	CCAATGGATCGGAAAATAAAG
*SbSUT6*	GCACAACAGCACAAAGAAGG	AGGCAGAAGAGGCTGAGATG
*SbGAPDH*	AGGGTATCATGGGCTACGTG	AGTTGTCGTTCAGGGCAATC
*SbEF1a*	CATGGTGGTGGAGACCTTCT	TCCTTCTTCTCCACGCTCTT

### YEAST TRANSFORMATION

*Sorghum* *SUT*-yeast expression vector constructs were introduced into the *Saccharomyces cerevisiae* yeast strain SEY6210 (MATα leu2–3, 112 ura3–52 his3–Δ200 trpl-Δ901 lys2–801 suc2- Δ9 GAL; Robinson et al., 1988) using the 40% PEG1000 transformation method (Dohmen et al., 1991). Yeast transformants harboring one of each of the cv. Rio SUTs, the cv. BTx623 SbSUT5 (SbSUT5G) and empty pDR196 vector were identified. Media lacking uracil was used for selection as the pDR yeast expression vectors contain the uracil synthesis gene. DNA was extracted from yeast post transformation, then plasmids were transformed into *Escherichia coli *(strain DH5α), and were harvested using a Plasmid Mini Kit (QIAGEN). Plasmids were sequenced to confirm that the *SUT* sequences were correct. In short, 1.5mL yeast culture was pelleted, washed with MilliQ water then resuspended in lysis buffer [50 mM Tris-HCl pH 8, 100mM NaCl, 1% SDS, 2% Triton X-100, 1mM ethylenediaminetetraacetic acid (EDTA)]. Glass beads were added (0.3g, 425–600 μm diameter) along with 200 μL phenol:chloroform:isoamyl alcohol (25:24:1; Sigma-Aldrich, Castle Hill, NSW, Australia) and vortexed for 10 min followed by micro-centrifugation for 5 min at maximum speed. The upper extract layer was then removed and DNA precipitated in 1 mL ethanol prior to pelleting and resuspension in 50 μL TE.

### YEAST COMPLEMENTATION

Transformed yeast strains harboring *Sorghum* SUTs, empty pDR196 and PsSUT1 were grown in liquid culture to an OD_600_ of 0.8 in synthetic dropout media lacking uracil. Untransformed yeast was cultured in synthetic complete media. Yeast were streaked (2 μL) on solid media lacking uracil and supplemented with either sucrose (25 mM) or glucose (100 mM) as the sole carbon source. This was repeated three times and plates were photographed using a ChemiDoc^TM^ XRS system (Bio-Rad, Gladesville, NSW, Australia). SuSy7 yeast harbouring PsSUT1-pDR196 was kindly provided by Zhou et al. (2007) for use as a positive control.

### *SUT* TRANSCRIPT QUANTIFICATION BY qPCR

Primers used for quantitative PCR (qPCR; **Table 1**) were designed to amplify regions of the 3′ UTR of each *SUT* due to high sequence homology within coding regions, with the exception of *SbSUT2* where a region from the coding sequence was amplified. Products from standard PCR were sequenced to ensure that correct gene fragments were amplified. Quantitative PCR was carried out on a Rotor-Gene Q (QIAGEN) using the QuantiFast SYBR green PCR kit (QIAGEN) and a two-step cycling program according to the manufacturer’s instructions. The green channel was used for data acquisition. Gene expression was measured relative to the housekeeper, *Sorghum bicolor elongation factor 1-alpha* (*SbEF-1α*).

### SELECTION OF HOUSEKEEPING GENE FOR qPCR

**FIGURE 1:**
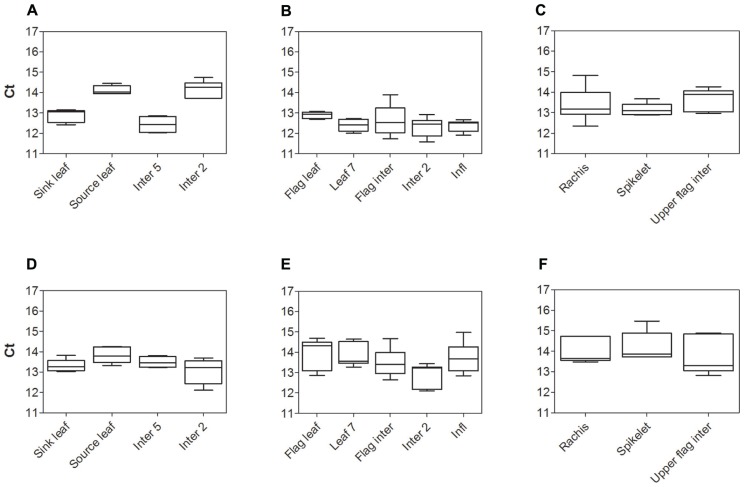
**Analysis of *Sorghum* housekeeping gene *SbEF-1α* expression, by qPCR.** Cycle threshold (Ct) values for the *Sorghum* *SbEF-1α* gene in cv. Rio **(A–C)** and cv. BTx623 **(D–F)** during vegetative growth **(A,D)**, at anthesis **(B, E)**, and within the upper flag internode and inflorescence components at anthesis **(C, F)**. Box and whisker plots represent minimum to maximum Ct value, with upper and lower quartile from five biological replicates.

The expression stability of two widely used housekeeping genes, *Sorghum bicolor glyceraldehyde-6-phosphate dehydrogenase* (*SbGAPDH*) and *SbEF-1α* from cv. Rio, were assessed prior to measuring expression levels of *Sorghum SUTs*. Comparison of cycle threshold values (Ct) and absolute expression levels (data not shown) revealed both housekeeping genes were quite stably expressed within each organ examined. However, differences in expression of *SbGAPDH* were greater than those for *SbEF-1α*. Hence *SbEF-1α *was chosen to normalize *SUT* expression in subsequent experiments. The stability of *SbEF-1α *was compared between cv. BTx623 and cv. Rio (**Figure 1**). Expression of *SbEF-1α *was least stable in cv. Rio during vegetative growth (Source leaf and Inter 2 – **Figure 1A**) and cv. BTx623 at anthesis (Inter 2 – **Figure 1E**). However, in all cases this variation was insignificant relative to the observed genotypic differences in the relative expression levels of the genes of interest and hence had no impact on the conclusions drawn.

## RESULTS

### SbSUT SEQUENCES

**FIGURE 2:**
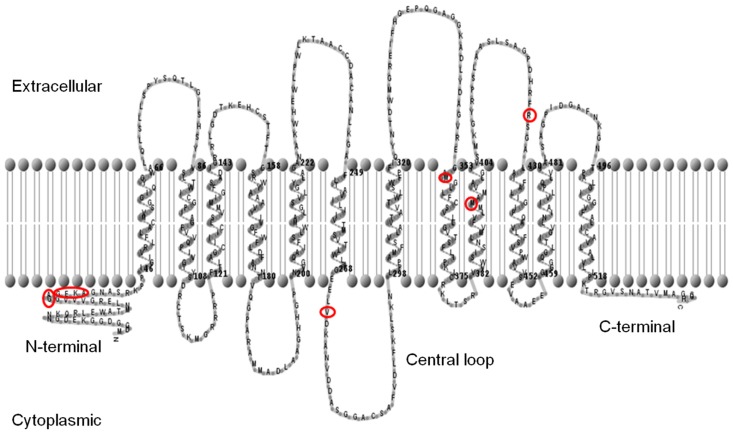
**Predicted membrane topology of the SbSUT5 protein.** The predicted trans-membrane regions of the SbSUT5 transporter from sweet *Sorghum* (Rio), identifying which amino acids differ between cv. Rio and cv. BTx623 (red circles). Twelve trans-membrane domains are predicted. The membrane topology was generated using the TMHMM server v 2.0 (http://www.cbs.dtu.dk/services/TMHMM/) and TMRPpres2D viewer (Spyropoulos et al.,2004).

Full-length coding sequences of each *SUT* from both *Sorghum* cultivars were amplified by PCR, cloned, and then sequenced. Twelve trans-membrane domains were predicted for each SUT using the TMHMM (Hidden Markov model-based transmembrane) predictive algorithm, and a graphical representation of the membrane topology of SbSUT5 is shown (**Figure 2**). Cytoplasmic N- and C-termini were predicted along with a central loop domain. Sequence analysis (not shown) revealed that a number of conserved features are present in *Sorghum* SUTs. A conserved histidine residue is present in the first loop domain corresponding to His-65 (Lu and Bush, 1998) and amino acids which correspond to the G-X-X-X-D/E-R/K-X-G-[X]-R/K-R/K motif reside in the second and eighth loop domains (Lemoine, 2000; Pazdernik et al., 2000). Only SbSUT4 contained an LXXLL motif in the N-terminal domain, indicating it may be targeted to the tonoplast (Yamada et al., 2010).

A number of amino acid differences were noted between cv. Rio *SUTs* and the published cv. BTx623 genomic sequence. To examine this further, *SUTs* from cv. BTx623 were cloned and sequences verified. SbSUT1 and SbSUT2 possessed single amino acid sequence differences, whereas SbSUT3, SbSUT4, and SbSUT6 were identical when sequences from cv. Rio and cv. BTx623 were aligned. SbSUT1 from cv. Rio had a valine (V) at position 381, whereas cv. BTx623 had an isoleucine (I) in this position. In SbSUT2 at amino acid 41, a threonine (T) was present in the sequence from cv. Rio, but absent in the cv. BTx623 sequence. SbSUT5 exhibited the most variation between the two cultivars with nine amino acid differences. Five amino acids out of a string of six differed between cv. BTx623 and cv. Rio SUT5, and were predicted to lie in the N-terminal region of the transporter (**Figure 2**). Starting at amino acid 32, the cv. Rio sequence predicted GAGEKA whilst the cv. BTx623 sequence predicted AGEKKG. Single amino acid differences between the cv. Rio and cv. BTx623 sequences occurred at amino acid 272, 355, 396, and 426 as shown in **Figure 2** (V272L; M355V; M396T, and R426K, respectively). These amino acid sequence differences in the SUTs between the two cultivars are summarized in **Table 2**.

**Table 2:** Summary of SUT sequence variation between BTx623 and Rio cultivars.

SUT	No. of variations (BTx623 vs Rio)	Amino acid variations
SbSUT1	1	I381V
SbSUT2	1	T41 insertion (Rio)
SbSUT3	0	–
SbSUT4	0	–
SbSUT5	9	A32G, G33A, E34G, K35E, G37A,L272V, V355M, T396M, K426R
SbSUT6	0	–

### PHYLOGENETIC ANALYSIS OF MONOCOTYLEDONOUS SUTs

**FIGURE 3:**
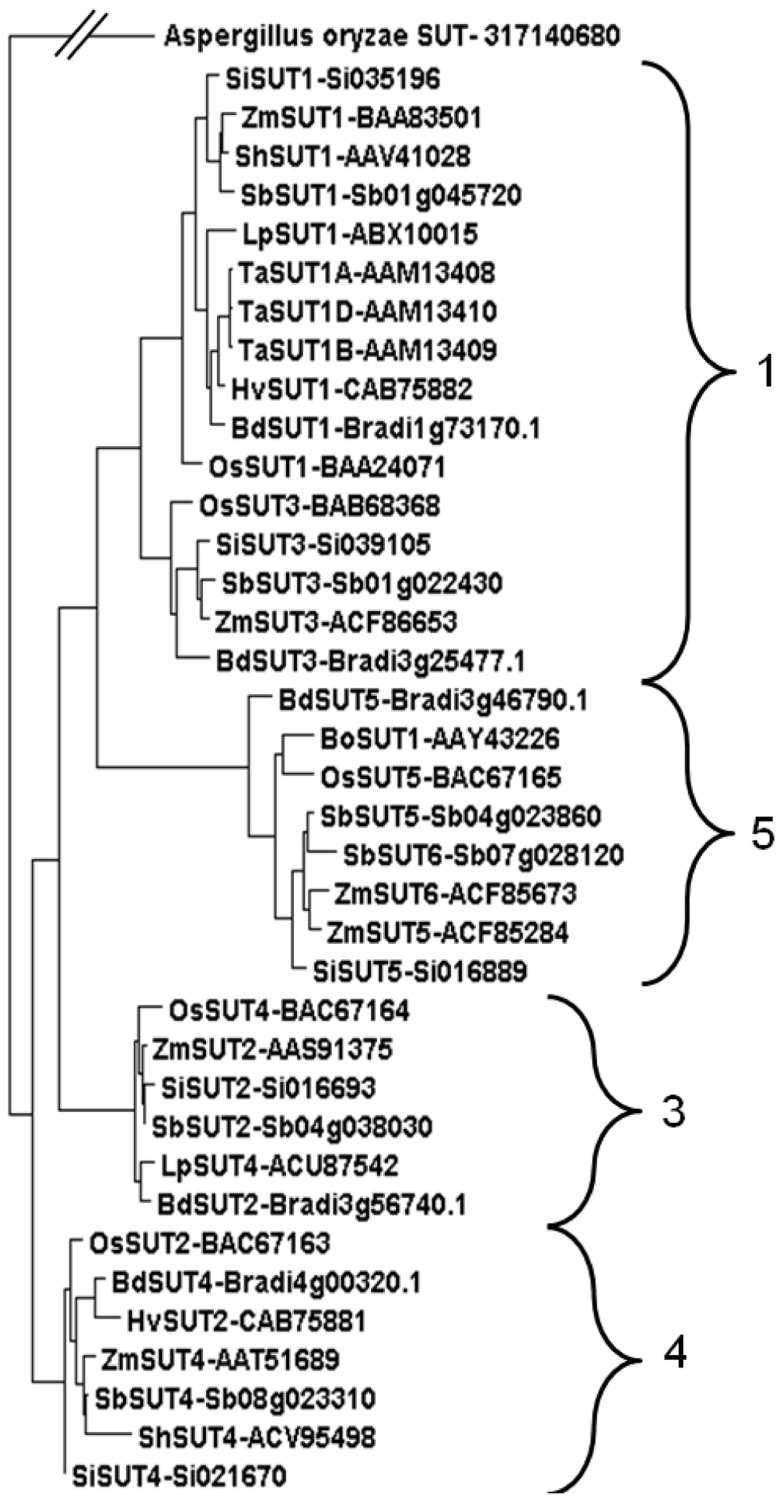
**Phylogenetic analysis of SUTs from monocotyledonous species.** SUTs displayed fit into Groups 1, 3, 4, 5 (Braun and Slewinski, 2009) from species Brachypodium distachyon* (BdSUT1, BdSUT2, BdSUT3, BdSUT4, BdSUT5), *Bambusa oldhamii* (BoSUT1), *Hordeum vulgare* (HvSUT1, HvSUT2), *Lolium perenne* (LpSUT1, LpSUT4), Oryza sativa* (OsSUT1, OsSUT2, OsSUT3, OsSUT4, OsSUT5), *Saccharum* hybrid (ShSUT1, ShSUT4), Setaria italica* (SiSUT1, SiSUT2, SiSUT3, SiSUT4, SiSUT5), Sorghum bicolor* (SbSUT1 – Sb01g045720, SbSUT2 – Sb04g038030, SbSUT3 – Sb01g022430, SbSUT4 – Sb08g023310, SbSUT5 – Sb04g023860, SbSUT6 – Sb07g028120), *Triticum aestivum* (TaSUT1A, TaSUT1B, TaSUT1D), Zea mays* (ZmSUT1, ZmSUT2, ZmSUT3, ZmSUT4, ZmSUT5, ZmSUT6). Phylogenetic analysis was carried out using MUSCLE alignment, Gblocks curation followed by PhyML phylogeny (Dereeper et al., 2008) before viewing in Dendroscope (Huson et al., 2007). Accession numbers are shown along with gene identifications (*Brachypodium*, *Setaria*, and *Sorghum*). Asterisks indicate that the full genomic sequence is publicly available.

A phylogenetic analysis demonstrated that the *Sorghum* SUTs clustered into four clear groups (**Figure 3**). This is consistent with phylogenetic analyses of other grass species including the C_3_, *Lolium perenne* (Berthier et al., 2009) and the C_4_ *Zea mays *(Braun and Slewinski, 2009). Two transporters appeared in Groups 1 and 5. In previous studies, Group 2 contained only SUTs from eudicots (Berthier et al., 2009; Braun and Slewinski, 2009). The *Sorghum* SUTs aligned closely with SUTs from other C_4_ monocotyledonous species such as maize, sugarcane, and *Setaria viridis* (**Figure 3**).

### EXPRESSION OF *SUTs* IN YEAST

**FIGURE 4:**
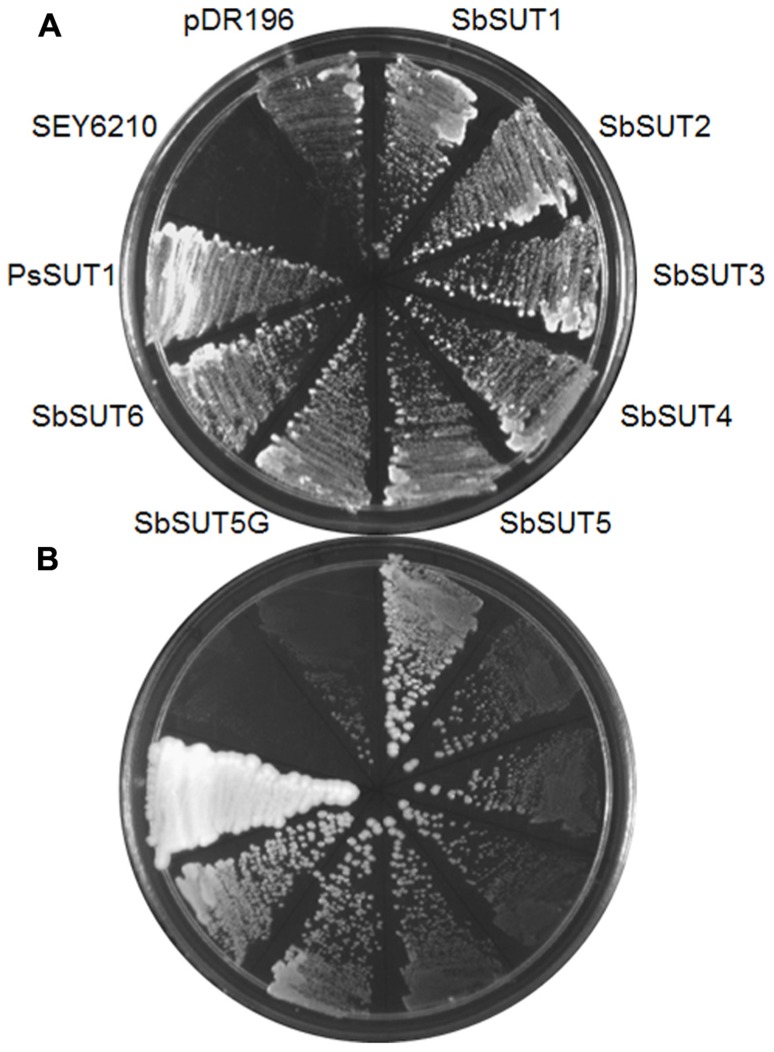
**Complementation of the SEY6210 yeast strain by *Sorghum* SUTs.** All *Sorghum* SUTs were expressed in the yeast strain SEY6210 and grown on media containing **(A)** 100 mM glucose or **(B)** 25 mM sucrose as the sole carbon source. SuSy7 containing PsSUT1 was used as a positive control. The SuSy7 PsSUT1-pDR195 (Zhou et al., 2007) was used as a positive control and negative controls were untransformed SEY6210 and pDR196 empty vector.****

*SUTs* from cv. Rio were cloned and expressed in yeast using pDR195 or pDR196 yeast expression vectors (Rentsch et al., 1995), along with the *SbSUT5* from cv. BTx623. The SEY6210 strain of *Saccharomyces cerevisiae* supported growth on media containing sucrose as the sole carbon source, when complemented with each *SUT* (**Figure 4**). This indicates that the introduced SUT mediated sucrose import from the media to support yeast growth.

### TRANSCRIPT LEVELS OF *SUTs*

**FIGURE 5:**
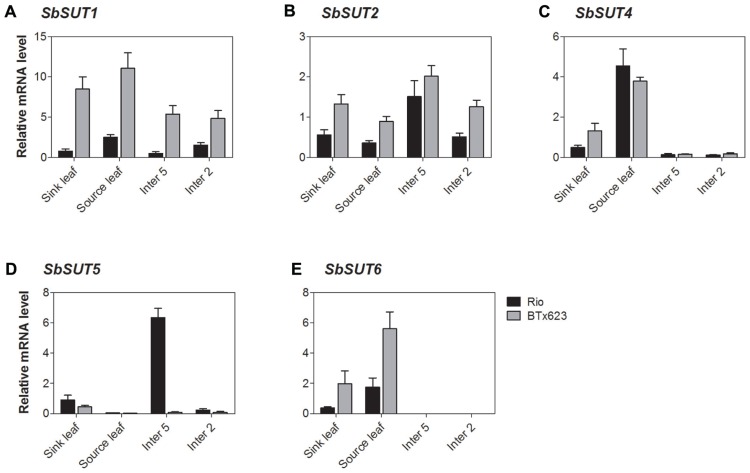
***Sorghum* SUT transcript levels during vegetative growth.** Relative expression during vegetative growth of *Sorghum* SUTs. **(A)** *SbSUT1*; **(B)** *SbSUT2*; **(C)** *SbSUT4*; **(D)** *SbSUT5*; **(E)** *SbSUT6*. Levels of *SUT* expression were measured relative to *SbEF-1α*. Organs examined were a Sink leaf (expanding); Source leaf (youngest fully expanded); Internode 5 (Inter 5, elongating); and Internode 2 (Inter 2, fully elongated). Columns with vertical bars represent mean ± SE from five biological replicates.

**FIGURE 6:**
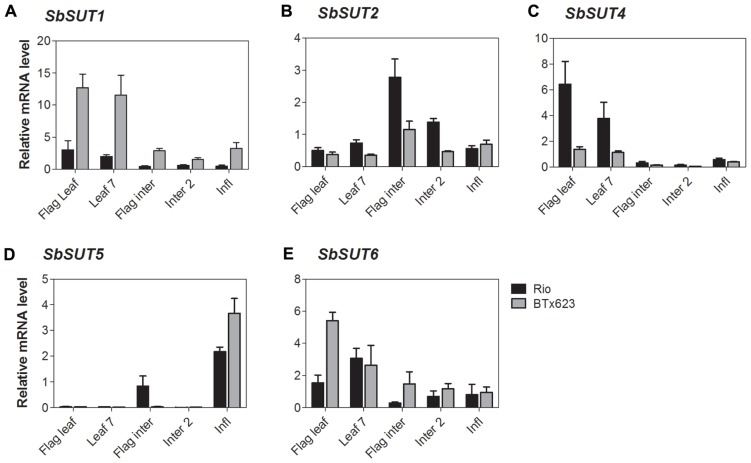
***Sorghum* SUT transcript levels at anthesis.**Relative expression at anthesis of *Sorghum* SUTs **(A)** *SbSUT1*; **(B)** *SbSUT2*; **(C)** *SbSUT4*; **(D)** *SbSUT5*; **(E)** *SbSUT6*. Levels of *SUT* expression were measured relative to *SbEF-1α*. Organs examined were the Flag leaf; Leaf 7; flag internode (Flag inter); Internode 2 (Inter 2) and the inflorescence (Infl). Columns with vertical bars represent mean ± SE from five biological replicates.

**FIGURE 7:**
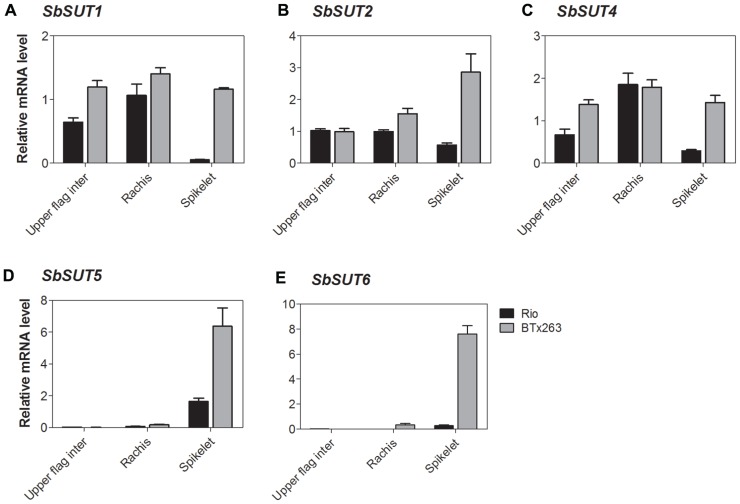
***Sorghum* SUT transcript levels at anthesis in the upper portion of the flag internode and within the inflorescence.** Relative expression at anthesis of the *Sorghum* SUTs **(A)** *SbSUT1*; **(B)** *SbSUT2*; **(C)** *SbSUT4*; **(D)** *SbSUT5*; **(E)** *SbSUT6*. Levels of *SUT* expression were measured relative to *SbEF-1α*. Organs examined were the Rachis and Spikelet as well as the upper portion of the flag internode (Upper flag inter). Columns with vertical bars represent mean ± SE from five biological replicates.

All *SUTs* were expressed at measurable levels in all organs examined apart from *SbSUT3*, consistent with previous observations (Qazi et al., 2012). *SbSUT1 *transcripts were detected in both source and sink organs with higher levels observed in cv. BTx623 compared to cv. Rio (two to threefold higher; **Figures 5A and 6A**). During the vegetative stage of development, fully expanded leaves exhibited the highest level of expression, followed by expanding leaves and stems (**Figure 5A**). At anthesis, fully expanded leaves exhibited substantially higher (fourfold) levels of expression than stems and inflorescences (**Figure 6A**). Expression levels were similar between cultivars in upper portions of their flag internodes along with rachis branches, but were greater in cv. BTx623 than cv. Rio in spikelets (**Figure 7A**).*SbSUT2 *was expressed in all organs examined in both cultivars. During vegetative growth, expression was slightly higher in young elongating stems compared to other organs (**Figure 5B**). At anthesis, flag internodes had the highest levels of *SbSUT2* transcript with cv. Rio being twofold higher than cv. BTx623 (**Figure 6B**). Transcript levels were highest in the cv. BTx623 spikelets exhibiting a threefold difference compared to cv. Rio. Twofold higher levels of expression were observed in spikelets of cv. BTx623 than in rachis branches and upper portions of flag internodes of either cultivar (**Figure 7B**).

During vegetative growth, *SbSUT4 *exhibited a similar pattern of expression in the two cultivars. *SbSUT4 *expression was highest in fully expanded leaves, with at least twofold lower levels in other organs examined and especially so for stems (**Figure 5C**). In contrast, at anthesis, transcript levels of *SbSUT4* in source leaves were two to threefold greater in cv. Rio compared to cv. BTx623. Within inflorescences, *SbSUT4* transcripts were equally high in rachis branches but for spikelets, expression levels in cv. BTx623 exceeded those of cv. Rio by fourfold (**Figure 7C**). The cultivar difference was reflected, but to a lesser extent, in upper portions of their flag internodes (**Figure 7C**).

There was a clear trend in *SbSUT5* expression during the vegetative stage of development and at anthesis, and expression levels differed between cv. Rio and cv. BTx623 at both developmental stages. During vegetative growth, *SbSUT5 *was strongly and exclusively expressed in elongating Internode 5 of cv. Rio (**Figure 5D**). At anthesis, the dominant level of expression switched to inflorescences with cv. BTx623 expression levels exceeding those of cv. Rio by ca 50% (**Figure 6D**). Within inflorescences, *SbSUT5* was expressed primarily in spikelets with threefold higher levels in cv. BTx623 compared to cv. Rio (**Figure 7D**). Transcripts were present in the flag internode of cv. Rio and absent in the same organ of cv. BTx623 (**Figure 6D**).

Transcripts of *SbSUT6* were only detected in sink and source leaves during vegetative growth with levels in cv. BTx623 being threefold greater than those of cv. Rio (**Figure 5E**). At anthesis, leaf expression dominance was retained with cultivar differences declining with leaf age (compare flag and leaf 7 – **Figure 6E**). However, low transcript levels were detected in stems and inflorescences (**Figure 6E**). Within inflorescences, *SbSUT6* was strongly expressed in cv. BTx623 spikelets and either weakly expressed or absent from rachis branches and upper portions of flag internodes (**Figure 7E**).

## DISCUSSION

The full genomic sequence of *Sorghum* has allowed identification of all *SbSUT* sequences in this model cereal monocot. Examination of *SbSUT *transcript levels in source leaves versus stem and inflorescence sinks provides a strong indication of the role each transporter may play in transporting sucrose from source leaves to these sinks. To further highlight these roles, two phenotypically different cultivars were used, BTx623 and Rio. BTx623 preferentially partitions sucrose to developing inflorescences and hence an emphasis on grain yield whilst cv. Rio stores sucrose in stem parenchyma cells similar to sugarcane. Collectively these analyzes begin to identify which SUTs may participate in phloem loading, axial phloem transport, and phloem unloading.

All six SbSUTs demonstrated complementation of the deficient yeast strain, SEY6210 (**Figure 4**), indicating they are sucrose transport competent, and likely to be functional *in planta*. The single amino acid sequence differences in SbSUT2 sequence between cultivars is predicted to lie in its N-terminal domain, as does the string of amino acids which vary in the SbSUT5 (see **Figure 2** and**Table 2**). The N-terminal domain has been shown to alter SUT affinity for sucrose (Schulze et al., 2000). In addition, recent evidence has identified particular amino acids in rice SUT1 which alters its transport activity (Reinders et al., 2012; Sun et al., 2012). However, these do not appear to correspond with amino acid differences we have identified between cv. BTx623 and cv. Rio (**Figure 2** and **Table 2**). Possible impacts of the detected SbSUT sequence differences between cultivars observed here need to be assessed experimentally.

**FIGURE 8:**
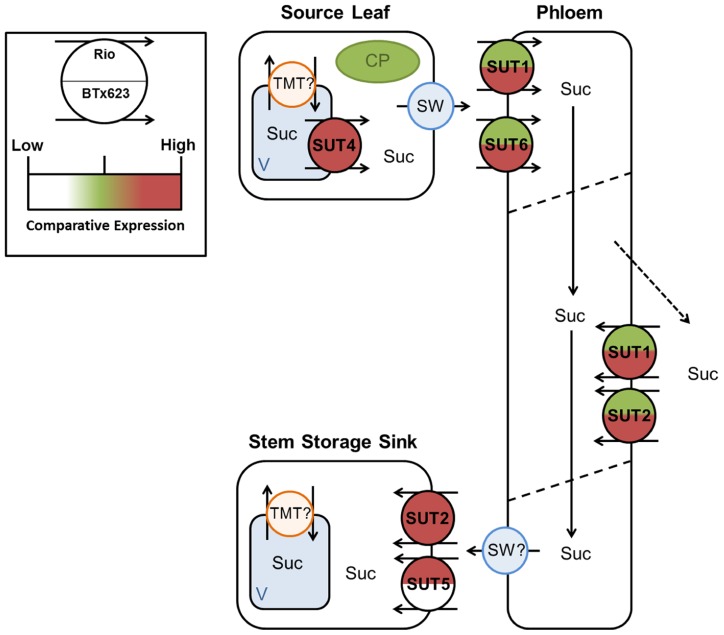
**Predicted source–transport–sink pathway in *Sorghum* during vegetative growth.** Sucrose is released from source vacuoles (V) by SbSUT4. SbSUT1 and SbSUT6 load the phloem. SbSUT1 and SbSUT2 may act to retrieve sucrose leaked from the transport phloem. SbSUT2 and SbSUT5 load sucrose into stem sinks. SWEETs (SW) efflux sucrose to the apoplasm and tonoplast monosaccharide transporters (TMT) move sucrose into vacuoles. Comparison of relative expression of each SUT is color coded.

**FIGURE 9:**
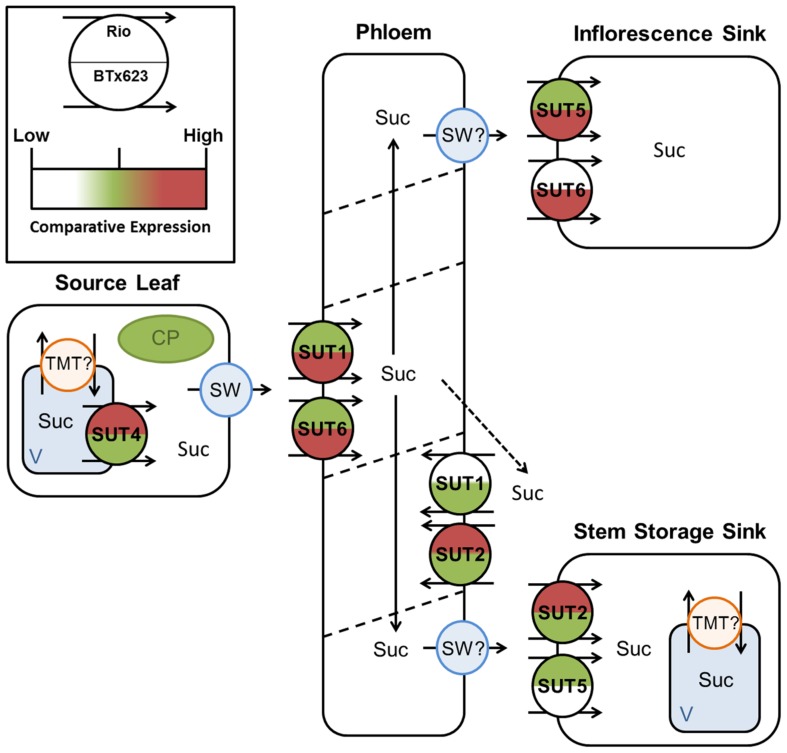
**Predicted source–transport–sink pathway in *Sorghum* at anthesis.**Sucrose is released from source vacuoles (V) by SbSUT4. SbSUT1 and SbSUT6 load the phloem. SbSUT1 and SbSUT2 may act to retrieve sucrose leaked from the transport phloem. SbSUT2 and SbSUT5 load sucrose into stem sinks. SbSUT5 and SbSUT6 load sucrose into reproductive sinks. SWEETs (SW) efflux sucrose to the apoplasm and tonoplast monosaccharide transporters (TMT) move sucrose into vacuoles. Comparison of relative expression of each SUT is color coded.

In terms of phloem loading, based on their relative expression levels in source leaves, identified SbSUT4, SbSUT1, and SbSUT6 as potential candidates during vegetative and reproductive growth (see **Figures 5, 6, 8, and 9**). For SbSUT4, this assertion is consistent with a high source leaf expression observed for *OsSUT2* (Eom et al., 2011) and *Populus tremula* × *alba *(gray poplar) *PtaSUT4 *(Payyavula et al., 2011). A number of transporters belonging to the same phylogenetic group as SbSUT4 (see **Figure 3**), have been localized to the tonoplast. These include rice SUT2 (Eom et al., 2011), barley SUT2 (Endler et al., 2006; Group 4) and the dicotyledonous SUTs, AtSUT4 (Endler et al., 2006), *Lotus japonicus* LjSUT4 (Reinders et al., 2008), poplar PtaSUT4 (Payyavula et al., 2011), and tobacco NtSUT4 (Okubo-Kurihara et al., 2011). On the tonoplast, SUT4 functions to release sucrose from mesophyll vacuoles to their cytoplasm (Schulz et al., 2011; Schneider et al., 2012) rendering the vacuolar pool of sucrose available for phloem loading. The significance of this function for SUT4 is demonstrated by slowed photoassimilate export in knock down SUT4 mutants of rice (Eom et al., 2011).

SbSUT1 may play a role in apoplasmic phloem loading (**Figures 8 and 9**) as found for the closely related maize ZmSUT1 which also belongs to the C_4_ NADP-ME subgroup (Slewinski et al., 2009). This assertion is based on finding that a maize *sut1* mutant exhibited a phenotype of shorter stature and carbohydrate accumulation in their source leaves (Slewinski et al., 2009). Consistent with this phenotype, the *sut1* mutant had a diminished ability to export sucrose from source leaves. Greater levels of *SbSUT1 *transcript were detected in source leaves of cv. BTx623 than cv. Rio (in apoplasmic phloem load **Figures 5A and 6A**). These differences could reflect differences in sink demand between cultivars driving photosynthetic rate along with sucrose export from source leaves (Minchin et al., 2002; McCormick et al., 2006). *SbSUT6 *is another phloem loading candidate (**Figures 8 and 9**). Similar to *SbSUT1*, transcript levels of *SbSUT6* were higher in source leaves of cv. BTx623 than cv. Rio at both vegetative and anthesis stages (**Figures 5E and 6E**) supporting the notion that sink demand might be stronger in cv. BTx623.

In sweet *Sorghum*, sucrose is radially transferred from the phloem into stem storage parenchyma cells through a post-sieve element unloading pathway that likely includes an apoplasmic step (Tarpley and Vietor, 2007). In this context, *SbSUT2 *and *SbSUT5* were highly expressed in internodes (**Figures 5B and 6B**) where they may play a primary role in phloem unloading, retrieval of sucrose leaked from the phloem or phloem loading of sucrose remobilized from stem storage (**Figures 8 and 9**). However, since remobilization predominantly occurs during grain filling (Gutjahr et al., 2013), at anthesis SUTs likely function to facilitate radial transport of sucrose to stem storage pools or in retrieving sucrose leaked from the phloem.

Twofold greater expression of *SbSUT2 *was observed in internodes of cv. Rio as opposed to those from cv. BTx623 at anthesis (**Figure 6D)**, suggesting this SUT may play an enhanced role in directing sucrose to stem storage parenchyma cells in cv. Rio. SbSUT2 has a predicted protein sequence of 594 amino acids, which is at least 60 amino acids longer than the five other *Sorghum* SUTs. Similarly, OsSUT4 has an extended central loop domain of around 90 amino acids and an extended N-terminal domain (Aoki et al., 2003). Little information is available for the other monocot Group 3 SUTs. However, an insertional mutant of the corresponding dicot SUT AtSUT3, showed no morphological phenotype (Barth et al., 2003).In comparison to the strong expression of *SbSUT5 *in cv. Rio internodes (**Figures 5D and 6D**), *SbSUT5 *transcripts were absent from cv. BTx623 internodes. Rather *SbSUT5* transcripts were 1.5–2-fold higher in inflorescences of cv. BTx623 than cv. Rio (**Figure 6D**), and especially spikelets (**Figure 7D**). These expression patterns suggest that cv. BTx623 directs more sucrose toward development of reproductive structures than cv. Rio. *SbSUT6*, most closely related to *SbSUT5* (**Figure 3**), was strongly expressed within cv. BTx623 spikelets (**Figure 7E**) and hence may also contribute to inflorescence development (**Figure 9**).

Cultivar differences in expression profiles of *SbSUT5* were accompanied by the highest number of amino acid difference in SbSUT5 sequences (9 amino acids, **Table 2**). Little information is available about transporters belonging to Group 5 apart from *OsSUT5*. This gene exhibited broad expression across source and sink leaves as well as in developing grains of rice (Aoki et al., 2003). Similar to *SbSUT5*, SUT1 transporters of more distantly related C_3_ species of rice, barley, and wheat appear to play a role in grain development. Antisense lines for *OsSUT1* showed little phenotypic difference when compared to wild-type in vegetative growth or differences in carbohydrate content of their source leaves (Ishimaru et al., 2001). However, grain filling was reduced substantially in *OsSUT1* antisense plants (Scofield et al., 2002). A number of other monocotyledonous plant SUT1 transporters also were found to be involved in grain development, including barley (Weschke et al., 2000) and wheat (Aoki et al., 2002) SUT1-type proteins. Whether *SbSUT1* plays a major role in grain development remains to be determined.

In conclusion, the six *Sorghum SUTs* were cloned from two cultivars that differ in carbohydrate partitioning. Expression analysis revealed that three of the *SUTs* were expressed strongly in source leaves (*SbSUT1*, *SbSUT4*, *SbSUT6*) and are likely to play roles in phloem loading. Two *SUTs* were expressed strongly in sinks (*SbSUT2*, *SbSUT5*) and are more likely to play roles in sink development and photoassimilate storage. *SbSUT3* was not detected in most organs examined. All of the *Sorghum* SUTs complemented the deficient yeast system, indicating they are sucrose transport competent. A number of amino acid sequence variations were identified between the SUTs from the two cultivars, and future functional characterization will determine if these variations result in alteration of their sucrose transport properties.

## Conflict of Interest Statement

The authors declare that the research was conducted in the absence of any commercial or financial relationships that could be construed as a potential conflict of interest.
